# Enhancing health literacy through co-design: development of culturally appropriate materials on genetic risk and customary consanguineous marriage

**DOI:** 10.1017/S1463423618000038

**Published:** 2018-04-12

**Authors:** Parveen Azam Ali, Sarah Salway, Elizabeth Such, Andrew Dearden, Matt Willox

**Affiliations:** 1 Lecturer, The School of Nursing and Midwifery, University of Sheffield, Sheffield, UK; 2 Professor of Public Health, Health Equity & Inclusion Research Group, School of Health & Related Research, University of Sheffield, Sheffield, UK; 3 Research Fellow, Health Equity & Inclusion Research Group, School of Health & Related Research, University of Sheffield, Sheffield, UK; 4 Professor of Interactive Systems Design, Cultural Communication and Computing Research Institute (C3RI), Sheffield Hallam University, Sheffield, UK; 5 Design Researcher, Design Futures, Sheffield Hallam University, Sheffield, UK

**Keywords:** co-design, consanguinity, cousin marriage, ethnicity, genetic risk, health equity, health literacy, migrant, participatory

## Abstract

**Aim:**

To develop a simple health literacy intervention aimed at supporting informed reproductive choice among members of UK communities practising customary consanguineous marriage.

**Background:**

The contribution of ‘health literacy’ to reducing health inequalities and improving primary health-care efficiency is increasingly recognised. Enhancing genetic literacy has received particular attention recently. Consanguineous marriage is customarily practised among some UK minority ethnic communities and carries some increased risk of recessive genetic disorders among offspring compared with unions among unrelated partners. The need to enhance genetic literacy on this issue has been highlighted, but no national response has ensued. Instead, a range of undocumented local responses are emerging. Important knowledge gaps remain regarding how the development and implementation of culturally appropriate, effective and sustainable responses can be achieved.

**Methods:**

Our co-design approach involved active participation by local people. Initial insight generation employed six focus group discussions and 14 individual interviews to describe current understandings and information needs. A total of 11 personas (heuristic narrative portraits of community ‘segments’) resulted; four participatory workshops provided further understanding of: preferred information channels; feasible information conveyance; and responses to existing materials. Prototype information resources were then developed and feedback gathered via two workshops. Following further refinement, final feedback from health-care professionals and community members ensured accuracy and appropriateness.

**Findings:**

The project demonstrated the utility of co-design for addressing an issue often considered complex and sensitive. With careful planning and orchestration, active participation by diverse community members was achieved. Key learning included: the importance of establishing trusting and respectful relationships; responding to diversity within the community; and engendering a creative and enjoyable experience. The resultant materials were heavily shaped by local involvement. Evaluative work is now needed to assess impacts on knowledge and service uptake. Longer term sustainability will depend on whether innovative community-level work is accompanied by broader strategy including investment in services and professional development.

## Introduction

The importance of ‘health literacy’ in primary care is increasingly recognised (Rowlands and Protheroe, 2012; Rowlands *et al*., 2014). Low levels of health literacy are associated with poorer access to health services, poorer communication with health-care professionals, lower adherence to treatment and poorer self-management of health conditions. Improved health literacy could therefore contribute to reducing health inequalities and improving health-care efficiency (Bostock and Steptoe, 2012; Rowlands and Protheroe, 2012).

From early foundations (Simonds, 1974), the concept of health literacy has evolved to encapsulate a range of competencies that enable individuals to both understand and take action on the factors influencing their health (Sørensen *et al*., 2012). Health literacy depends not only on cognitive abilities, but also the accessibility, quality, timing, relevance and trustworthiness of information on offer, as well as the context within which it is provided (Sørensen *et al*., 2012). While clinical models of health literacy tend to focus on functional literacy (i.e., basic reading and numeracy skills), community health models recognise the interdependencies between individual and social determinants and emphasise the empowerment of individuals to overcome structural barriers to health (Nutbeam, 2000). Patients from minority ethnic backgrounds, particularly those with little education and high levels of deprivation, have been found to have poor health literacy in relation to a range of health issues and across varied settings (Lutfiyya *et al*., 2008; Vida Estacio *et al*., 2015). The need for locally sensitive, culturally appropriate, community-based and empowering approaches to enhancing health literacy has been emphasised (Nutbeam, 2000; Vida Estacio *et al*., 2015).

Enhancing health literacy in relation to the use of genetic risk information, and the role of primary care practitioners in this agenda, have received particular attention in recent years in the United Kingdom (Department of Health, 2003; Qureshi and Kai, 2008; Lea *et al*., 2011; Vassy *et al*., 2012). The present paper is concerned with the increased genetic risk associated with close blood relative (consanguineous) marriage. Overall, 10% of marriages, around the world, are between cousins. Consanguineous marriage is a socially acceptable practice in many countries such as the Middle East, Pakistan, Bangladesh, India, Turkey and Lebanon (Othman and Saadat, 2009). In the United Kingdom, cousin marriage is found occasionally among the majority White British population, but is more common, and often preferred, among a number of minority ethnic populations; the largest being those who identify as ‘Pakistani’ or ‘British Pakistani’ (Khan *et al*., 2016; Salway *et al*., 2016).

Consanguineous marriage increases the risk of recessive genetic disorders among offspring. Most people carry several gene mutations that do not affect their own health, but that can potentially cause a recessive disorder in their offspring (Speicher, Antonarakis and Motulsky, 2010). When both partners happen to carry the same recessive gene, each child has a one in four chance of inheriting it from both parents and therefore having the recessive disorder. At the population level, studies in a variety of settings suggest that the risk of any congenital anomaly is roughly doubled among populations practising customary close relative marriage – from around 3% to around 6% of births – (Bundey and Alam, 1992; Stoltenberg *et al*., 1997; Sheridan *et al*., 2013), though accurate estimates are compromised by unconfirmed diagnoses and pregnancy terminations. This higher risk is translated into higher infant mortality rates and increased prevalence of long-term health conditions and disabilities (Bittles, 2012). Interest in this issue has grown in recent years in England and other European countries that are home to significant minority ethnic populations of Asian and Arab origin. Notwithstanding considerable debate regarding the appropriate policy and practice responses (Stoltenberg *et al*., 1997; Department of Health, 2012; Hamamy, 2012; Teeuw *et al*., 2014; Shaw and Raz, 2015), the World Health Organisation (Alwan *et al*., 1997) and UK national experts (Modell and Darr, 2002; Darr *et al*., 2016) recommend combining community-level activity to enhance genetic health literacy with improved access to genetic services and health professional training to improve both genetic knowledge and understanding of sociocultural context.

Despite growing recognition of the need to address this health need, to-date, no national policy statements, service templates or standards have been produced in the United Kingdom. Instead, varied local responses are emerging, many of which include initiatives aimed at enhancing genetic literacy among community members and patients (Darr *et al*., 2016; Salway *et al*., 2016). However, so far the development of these interventions has not been documented and no formal evaluations of their implementation or impact have been reported. Clearly, health literacy approaches in this area remain in their infancy in England and elsewhere in Europe, and important knowledge gaps remain regarding how culturally appropriate, effective and sustainable responses can be put into practice. The present paper contributes to filling this gap by reporting on the development of a simple health literacy intervention.

## Background to the development

The development reported on here was commissioned by the public health department of a Primary Care Trust in the North of England in 2012. Local data indicated an infant mortality rate above the national average and large inequalities between ethnic groups. Health service planners and providers in the locality also identified lifelong disability linked to recessive genetic conditions and consanguinity as a concern (with estimates suggesting around 18 such births per year in the locality). Existing local intelligence suggested that levels of knowledge about the genetic risk associated with consanguineous marriage were low and also that there was wariness among local communities around discussing the topic of cousin marriage. Furthermore, the commissioning team were aware that professional stigmatisation of cousin marriages, insensitive media publicity and poorly devised past interventions in other parts of the country had resulted in a significant community backlash. The broader goal of the work was therefore to enable people to be aware of potential health risks and the choices available to them and not to chastise people for their cultural practices and marriage choices.

The development work had two aims: first to undertake insight work at community level, and second to develop and produce culturally appropriate information resources that reflected the needs of the community. The target population was identified as those who identified themselves as Pakistani or British Pakistani. The intention was that the health literacy initiative would raise awareness and understanding of the issues among local Pakistani people and prompt those who needed more information about their individual circumstances to visit their general medical practitioner (GP), who could then refer them on to specialist genetic services, if appropriate. An important consideration was the cost and sustainability of the initiative and so the desire was for resources that were standalone, for example leaflets, posters or audio materials and that did not require a health-care professional or other worker for delivery.

## Methods

### The development approach

This contextual background suggested the value of employing a co-design approach to the project. Co-design, like co-production, is founded on the principle that service users and citizens are experts in their own circumstances and it involves their active contribution to the design of services or interventions (Sanders and Stappers, 2008; Realpe and Wallace, 2010). Co-design challenges the differentiation of ‘expert’ from ‘lay’ knowledge, aims to draw on diverse perspectives and integrates the creativity of designers and people not formally trained in design (Sanders and Stappers, 2008). The form and extent of participation may vary within approaches that are labelled as co-design. However, co-design is distinguished from ‘consultation’ by actively involving end-users throughout the design process from problem definition, through ideas generation, development, testing and final production (Sanders and Simons, 2009). The arguments for co-design in health and social care are both pragmatic – user participation results in more appropriate designs – and moral – people have a right to be involved in design processes that have implications for their lives.

This type of approach can be particularly important when addressing populations who are poorly served or where understanding and trust between ‘professionals’ and citizens is lacking. Co-design can help to ensure that interventions – including those aimed at enhancing health literacy – reflect the diverse needs, values and wishes of different population groups. For example, Burke *et al*. (2004) co-designed health education materials with Vietnamese-American community members in Seattle on the topic of Hepatitis B prevention, screening and treatment. Their study highlighted the utility of ‘coalitions’ that bring together local people, community service organisations and health-care providers. These coalitions ensured that education materials integrated important elements, such as beliefs about traditional medicine and added legitimacy to the initiative.

Our team was made up of a multi-lingual nurse researcher, a public health researcher, two designers with expertise in participatory design, an anthropologist, a product designer and a multi-lingual research assistant. We actively involved representatives from community organisations, individuals identified as ‘community leaders’, religious leaders and lay members of the community. Health professionals, including genetic counsellors and geneticists, were also key to the co-design process as ‘co-experts’ drawn in at appropriate points along the development path.

**Figure 1:**
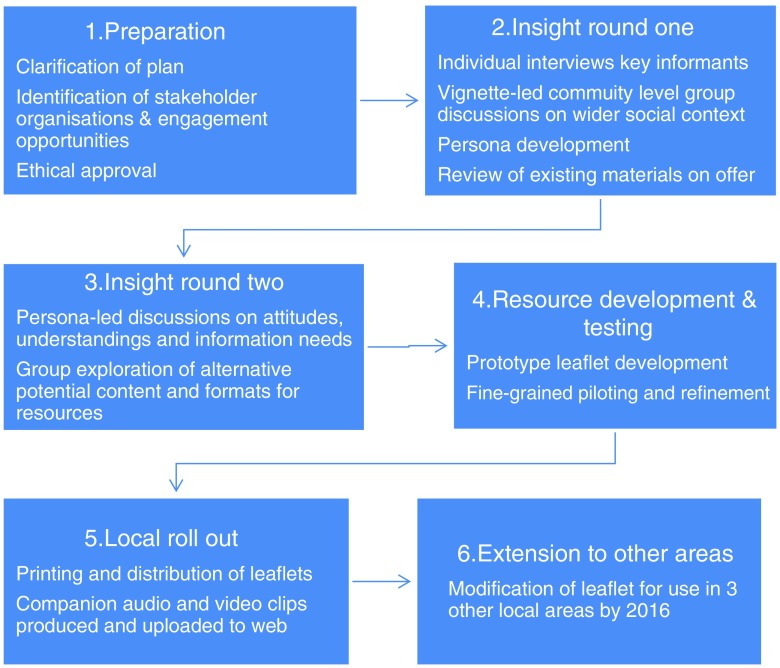
Project stages

We drew on ideas from three types of inquiry – social marketing, culturally competent participatory investigation and user-centred health-care design in order to design the principles, approach (Table 1) and stages of our development approach (Figure 1) (Wallerstein and Duran, 2010; Bowen *et al*., 2013; Das and Svanæs, 2013; Lefebvre, 2013; Morrison and Dearden, 2013).

**Table 1:** Summary of key co-design principles and approaches adopted

Principles	Approaches and techniques
1. Establish a participatory dialogue with community members (understanding and appreciating user perspectives, emotions, values and behaviours)	Diverse research team with language skills and cultural knowledge Use of familiar community venues Patient approach; time for trust-building Creation of welcoming and non-judgemental atmosphere Accommodation of participant needs (crèche; venues; timings) Requesting and respecting contributions throughout; active listening
2. Understand diversity within communities (segmentation) and challenge assumptions about the issues under investigation	Established links with a range of community-based organisations and varied points of contact with the community Engaged a diverse range of participants Used personas to explore diversity and challenge assumptions Negotiation of product design to meet diverse audience
3. Recognise structural constraints and facilitators to health literacy (including socio-political dimensions)	Open-ended, participatory exercises Exploration of health system factors and barriers to care
4. Positively innovate to find creative ways to overcome obstacles and seek new solutions to addressing the issue	Hands-on workshops using creative materials to try out varied content and formats Working across languages to interrogate understandings and find ways to convey meaning Team reflections
5. Material realisation – make tangible products with resonant messages and explore different strategies that can build on local resources	Iterative process with feedback on draft materials Identification of community venues and community workers interested to support material distribution Identification and reporting back of threats to sustainability

#### Preparation and insight work

Preparatory work involved developing networks with community organisations serving the needs of the local Pakistani population, developing the methodology for the work and gaining ethical approval. The first phase of insight work aimed to understand the context of cousin marriage through semi-structured individual interviews (*n*=14) and focus group discussions (*n*=6). Focus groups were single sex with four being conducted with men and two with women. Each group was attended by 8–14 participants with varied ages, education levels and English language competency. Participants were identified through local community organisations, English language classes and exercise groups. We did not specifically recruit participants on the basis of their marital status (whether unmarried, in a consanguineous union, or in a non-consanguineous union) nor their personal experience of genetic disorders, as we did not wish to stigmatise individuals or discourage participation. Furthermore, given our focus on developing resources aimed at raising general, community-level genetic literacy, rather than responding to information needs linked to particular individual or family-level genetic risk, a non-targeted approach was appropriate. However, once discussions were underway, participant contributions confirmed a range of current marital statuses and significant relevant experiences. Community leaders (*n*=4) (respected, high-profile members in the community including a mosque trustee, a manager of non-governmental organisation and community workers) and religious leaders/Imams (*n*=4) were also identified via community organisations and early workshops and recruited.

Interviews and focus group discussions were held in premises owned by or regularly used by community organisations. These both sought to establish the factors affecting marriage decisions such as the role of family members, the couple themselves and religious leaders. They also sought to explore participants’ awareness of genetic risk associated with close relative marriage and their level of awareness and use of available genetic services. To initiate discussion in both the interviews and the focus group discussions, an audio-recorded vignette – telling the story of a couple who became aware of their genetic condition and healthy carrier status following the birth of their baby affected by a recessive condition – was used. The recording was played and participants asked to listen. We then used a series of probing questions to gather further detail and elicit participant stories, opinions and experiences about cousin marriage and the associated genetic risk. A free-flowing, naturalistic discussion was encouraged. This approach facilitated discussion on the background to cousin marriage, the reasons for social practice and people’s own experiences in an open and non-judgmental environment. Discussions were held in English/Urdu/Punjabi according to participant preference and audio-recorded (subject to participant consent). Detailed notes were taken and a thematic analysis was performed. This analysis was then used to develop a series of 11 personas. These are heuristic devices that consist of narrative portraits encapsulating key traits of individuals in different ‘segments’ of the community and their relationship to the issue of focus (Cooper, 2003; Tod *et al*., 2012). The personas were designed to be credible and to characterise different people within the community and their divergent experiences and understandings (Tod *et al*., 2012). We also undertook a review of relevant publicly available health education resources. We identified and critically reviewed 30 information leaflets, booklets, websites and audio-visual material related to genetic disorders, consanguinity and recessive genetic disorders (26 in English; four in Urdu). We extracted from these ideas about possible content, language and terminology, layout and images.

**Figure 2:**
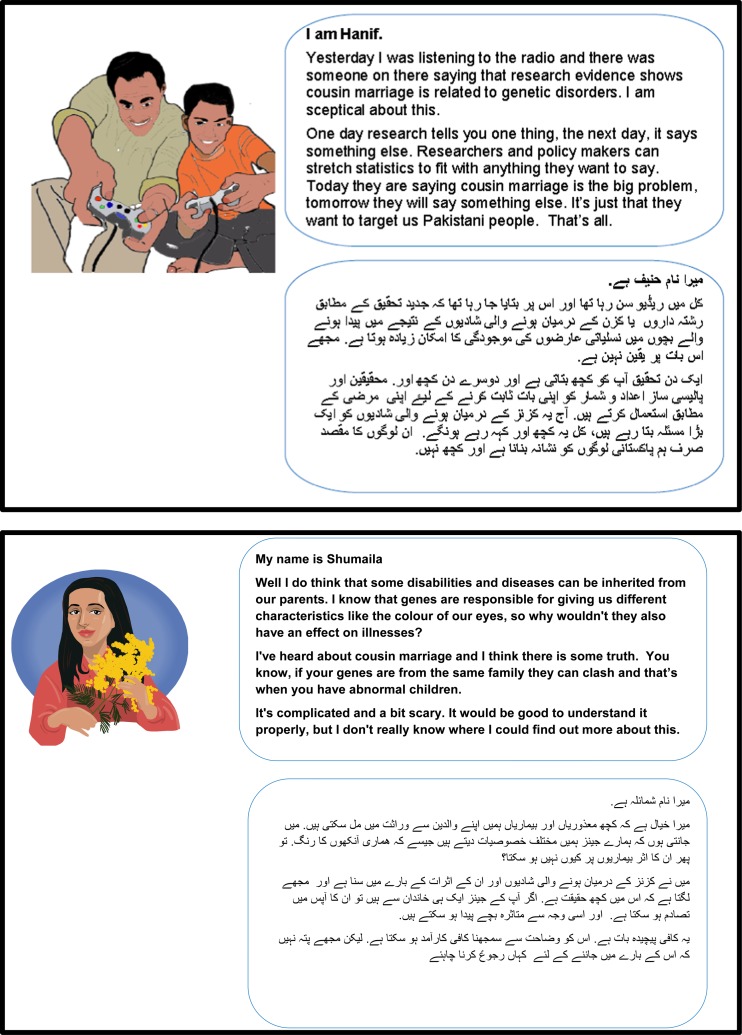
Contrasting personas

The insight gained from the above activities informed the subsequent round of insight generation in which we conducted four participatory workshops (one mixed sex and three single sex workshops). Each workshop was attended by 8–14 participants (with some participants carrying on from the first round and some new people joining the exercises). A key aim of the workshops was to extend insight into attitudes, knowledge and information needs. To verify understanding we produced the 11 personas on laminated on A3 size paper. Each persona contained a sketched image of a person, a name and a brief statement (in English and Urdu) demonstrating the individual’s point of view in relation to the issue at hand (Figure 2 provides an illustration of two of these contrasting personas). These posters were displayed around the room and used as a prompt for group discussion. Participants were asked to comment on whether the attitudes and circumstances represented in the personas were familiar to them and prevalent among their community members. The personas encouraged movement about the room, creating a lively atmosphere within which participants discussed the issues freely and also shared their own opinions and experiences.

In addition, these workshops aimed to generate further understanding of: preferred and trusted information sources; the feasibility of conveying information in different formats; and people’s responses to the ‘look and feel’ of varied communication materials. Drawing on pre-existing resources and a set of draft materials created by our product designer we presented workshop participants with a range of different media such as text, audio and video, and also alternative formats for example presenting statistics using pie charts, pictograms or in words. Workshops were facilitated by four team members and small group discussions were used to encourage active participation. Detailed notes were taken. Following each workshop, a team debriefing took place where the research team members reflected on the insight gained.

#### Resource development, testing and realisation

The next step was to develop prototype information resources. It was decided to initially develop printed leaflets. The leaflet was developed in six different layouts and sizes in English. The genetic information conveyed in the leaflet was developed during this phase with the help of experts in the field (one geneticist and two genetic counsellors). These prototype leaflets were then critically reviewed by the research team, the commissioning organisation and religious scholars. Two potential layouts, sizes and folding structure of information leaflet were selected to be taken forward to community-level testing; one a brief leaflet and the other a longer, more detailed version. The content of the leaflets was then translated into Urdu before being presented to community members in the next round of workshops.

Two workshops (one mixed sex and one single sex) were conducted. Each workshop was attended by 14–16 participants. The objective of these workshops was to obtain detailed feedback on the prototype resources in relation to their tone, adequacy, readability/comprehensibility, believability and acceptability. The research team and participants critically reviewed the leaflet line by line and engaged in detailed discussions to identify areas of further refinement. At this stage it became clear that a majority of participants felt that the briefer leaflet was not adequate and served to raise too many questions that it did not answer. Therefore, only the longer leaflet was taken forward to final production. After making a further round of changes to this leaflet, the research team sought final feedback from health-care professionals, genetic counsellors, geneticists and members of the community to ensure it was factually accurate and considered appropriate by this diverse group of actors.

### Considerations and justification for framing, content and format

The co-design process enabled the identification of key characteristics of the genetic literacy materials relating to overall framing, specific content and format (Table 2).

**Table 2:** Summary of design considerations identified and responses incorporated into the materials

Considerations	Responses
Framing	
1. Avoid stigmatising messages 2. Recognise the collective nature of reproductive decisions	Framed as ‘genetic conditions affect all communities’ Designed to be accessible to all age-groups and generations Encouragement to discuss and share with family members
Content	
1. Recognise mistrust of information sources 2. Address the need for moral and religious guidance 3. Recognise the complexity of the genetic information and the danger of contradiction if messages are too brief/simplistic	NHS logoFacts and figures with reference to trusted sources Moral/religious dimensions acknowledged Religious scholar contact information included Inheritance patterns carefully explained with pictorial illustration and words Encouragement to see the GP if concerned about genetic condition in the family
Format	
1. Use conceptual translation and pay attention to appropriateness of language 2. Consider dangers of stereotyping 3. Recognise diversity of learning styles within community	Consistent and familiar terms Ethnically ambiguous images of parents and baby Names that are common across ethnic/religious groups Variety of presentational approaches: factual; narrative; and question and answer

#### Framing

Co-designing an acceptable and legitimate information leaflet required careful attention to community members’ perspectives on cousin marriage, reproductive decision making and broader family contexts. A key message from the insight work was that these issues are usually collective issues. Workshop discussions with younger and older community members revealed that both groups recognised the importance of the other group in decision making and therefore it was important to develop information material general enough to suit the needs of different generations and to promote the leaflet as something to be shared and discussed with family members.

A further, general consideration of framing was that the leaflet should avoid singling out or stigmatising the Pakistani community. Workshop participants frequently challenged the focus on their community and noted the presence of genetic disorders and disabilities in other communities too. Participants stated that community members could feel negatively ‘targeted’. Participants therefore wanted the issues to be framed in the context of genetic risk being relevant to all communities. The leaflet included the statement ‘genetic disorders affect all communities’ and used an image that was ethnically ambiguous.

#### Content

Our co-design approach helped us to unearth the information needs recognised by members of the community rather than simply convey the information identified by health-care professionals as important. Three prominent issues were addressed in the content of the information resources. First, linked to the concern among some local people that their community was being targeted, blamed and stigmatised, there was a distrust of the claims made that the Pakistani population had a higher rate of birth anomalies than any other ethnic groups in the United Kingdom. Workshop participants called for the presentation of official statistics demonstrating the difference in the rates. To address this concern, we identified and included statistics from recognised sources to convey information on risks at the population as well individual level. Second, respondents raised the importance of considering the moral and religious aspects of the issue and wanted to have access to people who could advise on these dimensions to facilitate appropriate decisions. This required identification of reliable and trustworthy religious scholars who not only had the required knowledge about the issue and its religious and moral implications, but were also willing to be identified as contacts for the general public. Two such local religious scholars were identified and their contact details provided on the information resources. Third, the insight work highlighted the difficulties people had in understanding patterns of inheritance and the apparent inconsistencies they identified in information received via other sources. In particular, participants pointed out the contradiction they felt between the simplistic message: ‘your child is disabled because you are married to your cousin’, and the observation that couples could have both healthy and affected children. The leaflet needed to spell out clearly and completely how recessive disorders are inherited and the risks associated with each pregnancy.

#### Format

The co-design approach allowed us to experiment with the way the information was conveyed in the leaflets and seek feedback from a diverse range of participants to ensure comprehensibility and acceptability. This process identified the desirability of presenting information in different ways for different audiences within the community. While, as noted above, some individuals were keen to have facts and figures, others found a narrative style more engaging. The final design therefore incorporated both of these elements, with a brief story about one couple’s experience and a section including statistics. Furthermore, we synthesised a set of ‘frequently asked questions’ and included responses to these in the leaflet – an additional tailored way of conveying the core information.

Involving community members also helped to ensure use of appropriate and accessible language in both the English and Urdu versions of the information resources. Community members involved in the co-design exercises encouraged the use of conceptual, rather than literal, translation of the information from English into Urdu, and also identified when it was appropriate to retain those English words routinely used in Urdu conversation rather than using an uncommon Urdu equivalent. More generally, community members guarded against the use of language that was too formal or unfamiliar in the local context. Community members also guided the use of a particular font (*Noori Nustaleeq*) to ensure the information was understandable in Urdu.

Finally, in terms of format, the co-design approach provided important guidance to the ‘look and feel’ aspect of the leaflets. The exercises established the need for nuance in relation to the cultural appropriateness of the materials. Thus, while participants felt it was important to include information specific to the Pakistani population (as noted above), they cautioned against the use of images and names that could be seen to single out this group. Therefore, an image of parents with a baby and names were chosen that were ethnically ambiguous rather than specific.

### Implementation rollout

The use of a co-design approach was helpful in terms of putting in place linkages with community-based organisations and venues through which the resultant materials could be distributed. The engagement of community development workers in some aspects of the development process also meant that there were people working in these venues who understood the origin and purpose of the leaflets and were motivated to ensure they were accessible to local people. In the immediate period following the development work, 1000 leaflets were printed and distributed to 20 venues (including community organisations, mosques, GP surgeries) for display and distribution. Complementary audio and video materials that presented the same information were referenced in the leaflets and made accessible via a local NHS website link.

### Challenges and lessons learned

Team reflections undertaken during and after completion of the project identified four key challenges. First, negotiating access to community members via gatekeepers such as community leaders and health care professionals was time-consuming. We found that some such gatekeepers had a paternalistic attitude, seeking to protect local people from issues they felt would be contentious. Community leaders with no experience of genetic conditions in their family were particularly protective, and it was striking how differently most community members responded when given the opportunity to discuss the issues in an open and non-judgmental forum. In this regard, the role of bilingual researchers who self-identified as members of the Pakistani community, use of a patient, culturally sensitive approach and involvement of trusted community organisations were important strategies in gaining meaningful engagement. Over time, our use of a co-design approach helped to inculcate a sense of shared ownership of the information resources. Nevertheless, it should be acknowledged that not all segments of the community were sufficiently engaged, with teenagers and young adults, and working-age men, being groups that warrant further attention in future.

Second, this commissioned project had a very narrow remit and limited resources. The appropriateness of the objective to develop standalone materials – while the broad goal was one of enhancing genetic literacy and improving the uptake of genetics services – could be questioned. Indeed, the co-design exercises highlighted the varied information needs within the community and underscored the fact that the information being conveyed was complex and required multiple exposures for comprehension. Furthermore, for some individuals the opportunity to ask questions and discuss the issues was clearly important to developing new understanding. Within the context of this small project our response was to include within the leaflet encouragement to the reader to (i) share and discuss the information with family and friends and (ii) seek further information from the GP if concerned about a genetic condition in their own family. Ideally, however, the project would have had a broader remit and responded to WHO recommendations to develop a holistic approach to the issue, combining efforts to increase community-level genetic literacy with both enhanced genetics services and training to develop the necessary knowledge, skills and sensitivity among health-care professionals (Darr *et al*., 2013; Khan *et al*., 2016; Salway *et al*., 2016a). Indeed, our co-design workshops revealed some participants’ past experiences of receiving inaccurate information from health-care practitioners and difficulties in gaining referrals to the genetic service from primary care. There may be a danger therefore, that enhancing community genetic literacy alone could be ineffectual, or even possibly harmful, if concerns are raised without concomitant improvements to responses provided by the health system. Nevertheless, it should be noted that none of the people who engaged in the co-design exercises expressed the feeling that the development of the community health literacy materials was inappropriate or unhelpful.

Third, although our approach was iterative and allowed modification of the material in response to participant feedback on adequacy, comprehensibility and acceptability during the project period, it was not possible to seek subsequent feedback from community development workers on the reach or acceptability of leaflets as they were rolled out over time. Nor did the project include any formal assessment of changes in knowledge, attitude or behaviour resulting from exposure to the materials. Clearly, this type of summative evaluation of improvements in health literacy would be valuable in the future.

Finally, a further key challenge linked to the narrow focus of the innovation was the threat to sustainability. Commissioned by the public health team, the materials were intended to be distributed and promoted within primary care and community venues. However, the funding for the initiative was short-term and, following the initial round of distribution, it was unclear who would be responsible for monitoring their use and ensuring continued supply. On the other hand, the commissioner agreed to make the materials freely available and the research team took the initiative to acquire an appropriate Creative Commons licence and to publicise them across other regions with significant populations facing this health issue. As a result, the genetic literacy materials have been adapted for use in four other local areas in England so far and can be accessed via the internet (http://geneticsaware.group.shef.ac.uk/index.htm).

## Discussion

We have reported here on the development through co-design of a set of materials intended to enhance the health literacy of a British Pakistani community relating to the topic of close relative marriage and the risk of recessive genetic conditions. The project clearly demonstrated the feasibility and utility of a co-design approach to address an issue that has often been considered complex and sensitive. The project illustrated that with careful planning and orchestration, active participation in the development of the materials by a large and diverse group of community members over a series of iterative exercises was possible. Furthermore, the engagement of community members shaped the framing, content and format of the resultant materials in significant ways and resulted in a sense of shared ownership among participants.

There are few documented examples of health literacy development initiatives among minority ethnic communities and this project therefore contributes important new insights into how this type of work can be successfully undertaken. Key learning included the importance of establishing trusting and respectful relationships, recognising and responding to diversity within the community and engendering a creative and enjoyable experience. In this respect, our experiences chime with those reported in a recent review of co-creation approaches (Greenhalgh *et al*., 2016).

The project did, however, have a number of limitations. We have noted the limited input from teenagers and young adults to the co-design exercises, and would recommend exploring the potential of alternative contact points for these groups – such as through schools, colleges and youth groups and, possibly, via using social media – in any future similar projects. A second important shortcoming was the lack of any formal assessment of shifts in knowledge, attitudes or behaviours resulting from exposure to the materials. This is clearly something that should be assessed in a follow-on evaluation. A further particular concern was the focus on community members alone, with no attention being given to health-care professionals in primary care, or the genetic services on offer, as potential barriers to enhanced genetic literacy and better access to services. This latter point reminds us of the need to resist a very narrow, clinical model of health literacy and instead pay attention to the structural barriers to health (Nutbeam, 2000). As a commissioned research team charged with delivering a small-scale, time-bound project we were not in a position to re-draw the parameters of the work. Nevertheless, we made sure that the project report highlighted the limitations of the narrow, information-provision approach and encouraged the local policy-makers to consider a more holistic response moving forward. Finally, the lack of sustainability of the innovation was a concern. As discussed elsewhere, investments in ‘special’ initiatives that meet the needs of minority ethnic (and other relatively marginalised) populations are often short-lived unless they become integrated into mainstream provision, have strong national-level drivers and/or are championed by influential individuals (Salway *et al.*, 2016b).

In conclusion, this project confirms that co-design holds promise for developing more appropriate approaches to enhancing health literacy among minority ethnic people. We suggest that researchers and practitioners working on this or other aspects of health literacy might learn from the approaches described here. Further, we have highlighted a number of specific information needs that have previously been overlooked or poorly addressed in health literacy materials on the topic of genetic risk associated with consanguineous marriage. We hope that these will be responded to better in any future materials development. However, it is important to reiterate the message that an adequate response to this issue requires improvements to genetic service provision and enhanced understanding and competence among health-care professionals alongside increased genetic literacy at community level. Therefore, longer term impacts will depend on the extent to which such participatory work at community level is linked into policy-making and resource allocation structures that enable the necessary wider system changes ensue (Cacari-Stone *et al*., 2014).
